# Null and hypomorph *Prickle1* alleles in mice phenocopy human Robinow syndrome and disrupt signaling downstream of Wnt5a

**DOI:** 10.1242/bio.20148375

**Published:** 2014-09-04

**Authors:** Chunqiao Liu, Chen Lin, Chun Gao, Helen May-Simera, Anand Swaroop, Tiansen Li

**Affiliations:** 1 Neurobiology-Neurodegeneration and Repair Laboratory (N-NRL), MSC0610, 6 Center Drive, Bethesda, MD 20892, USA; 2 Imaging core facility, National Eye Institute, MSC0610, 6 Center Drive, Bethesda, MD 20892, USA; # Current address: The Ohio State University College of Medicine, 370 West 9th Avenue, Columbus, OH 43210, USA.

**Keywords:** Planar cell polarity, Development, Morphogenesis, Organogenesis, Conditional mouse mutants

## Abstract

Planar cell polarity (PCP) signaling plays a critical role in tissue morphogenesis. In mammals, disruption of three of the six “core PCP” components results in polarity-dependent defects with rotated cochlear hair cell stereocilia and open neural tube. We recently demonstrated a role of Prickle1, a core PCP molecule in *Drosophila*, in mammalian neuronal development. To examine *Prickle1* function along a broader developmental window, we generated three mutant alleles in mice. We show that the complete loss of *Prickle1* leads to systemic tissue outgrowth defects, aberrant cell organization and disruption of polarity machinery. Curiously, *Prickle1* mutants recapitulate the characteristic features of human Robinow syndrome and phenocopy mouse mutants with *Wnt5a* or *Ror2* gene defects, prompting us to explore an association of Prickle1 with the Wnt pathway. We show that Prickle1 is a proteasomal target of Wnt5a signaling and that Dvl2, a target of Wnt5a signaling, is misregulated in *Prickle1* mutants. Our studies implicate Prickle1 as a key component of the Wnt-signaling pathway and suggest that Prickle1 mediates some of the *WNT5A*-associated genetic defects in Robinow syndrome.

## INTRODUCTION

Embryonic development utilizes a network of transcription regulatory factors and signaling pathways to generate diverse cell types that are organized into functional entities (e.g., tissues). Within each tissue, individual cells require further instructions to establish an ordered architecture by acquiring specific polarity, such as front-rear polarity for migrating mesenchymal cells and apicobasal (AB) and planar cell polarity (PCP) for epithelial cells. Correct establishment of cell polarity is essential for organogenesis, tissue functions and survival.

PCP signaling provides vectorial information for cell arrangement and migration within a large field. First identified in *Drosophila*, the PCP module consists of six core components: Frizzled, Van Gogh/Strabismus, Dishevelled, Starry Night/Flamingo, Prickle, and Diego (Jenny and Mlodzik, 2006; Jenny et al., 2005; Seifert and Mlodzik, 2007; Wang and Nathans, 2007). In *Drosophila* wing epithelia, these proteins are segregated into two clusters within the apical domain, with Frizzled-Dishevelled-Diego-Flamingo localized distally, and Prickle-Vangl-Flamingo confined proximally (Jenny et al., 2005; Peng and Axelrod, 2012). A similar pattern is repeated in the neighboring cells and propagated throughout the morphogenetic field, offering directional information for hair positioning through cytoskeleton remodeling. The photoreceptors in *Drosophila* ommatidia also employ this protein segregation mechanism to facilitate local transmission of planar polarity information (Fanto and McNeill, 2004; Seifert and Mlodzik, 2007; Wang and Nathans, 2007; Zheng et al., 1995). Genetic disruption of PCP components in *Drosophila* leads to orientation defects in wing hair and ommatidia (Seifert and Mlodzik, 2007).

The PCP signaling pathway is evolutionally conserved. Each of the PCP components characterized in *Drosophila* has its homolog in vertebrates, and protein segregation and distribution pattern observed in Drosophila hair cells are also retained in some vertebrate epithelia (Deans et al., 2007; Vladar et al., 2012). Gene knockdown of PCP components in lower vertebrates results in defects in convergent-extension, ciliogenesis and neural tube closure (Wallingford, 2006; Wallingford et al., 2002; Wallingford and Harland, 2002). Disruption of PCP component genes in the mouse cause similar but more extensive tissue phenotypes, which include looped tail, abnormally rotated stereociliary bundles of the inner ear hair cells, and misoriented skin hair (Curtin et al., 2003; Hamblet et al., 2002; Montcouquiol et al., 2003; Tissir et al., 2010; Wang et al., 2006; Watanabe et al., 2003). Heart defects (Etheridge et al., 2008; Hamblet et al., 2002; Sinha et al., 2012; Yu et al., 2010) and kidney malformation (Simons et al., 2005; Tissir et al., 2010; Yates et al., 2010) have also been reported in PCP mutant mice.

Among the six core PCP components, the function of Prickle has remained largely elusive in mammals. The mouse genome has four *Prickle* homologs, with *Prickle1* and *Prickle2* being most closely related to their *Drosophila* prototype, whereas *Prickle3* and *Prickle4* are fairly distant. *Prickle1* and *Prickle2* were reported to be essential for AB polarity of embryonic epiblasts since ablation of either in mice resulted in early embryonic lethality prior to gastrulation (Tao et al., 2012; Tao et al., 2009). An ENU-induced frameshift mutation in mouse *Prickle1* resulted in limb defects and death shortly after birth (Yang et al., 2013), suggesting a yet to be explored late-stage Prickle1 function.

We recently demonstrated that *Prickle1* is expressed in the mammalian central nervous system and plays an important role in neuronal morphogenesis (Liu et al., 2013). To further explore a PCP-related function of Prickle1 at both early and late stages of development, we engineered three *Prickle1* mutant alleles in mice. Combinations of these with select Cre-drivers were predicted to generate varied gene-dosage in different tissues and bypass early embryonic lethality observed in *Prickle1* null mutants (Tao et al., 2009). Here, we report global and pleiotropic PCP defects in *Prickle1* null and hypomorphic mutants that closely resemble *Wnt5a*, *Ror* or *Ryk* mutant mice and recapitulate features of human Robinow syndrome (RS; RRS, MIM#268310, http://omim.org/entry/268310; DRS, MIM#180700, http://omim.org/entry/180700), prompting us to explore the link between Prickle1 and the Wnt pathway. Our studies implicate Prickle1 in regulating Wnt/PCP pathway and provide novel insights into RS disease mechanism.

## RESULTS

### *Prickle1* mutant alleles in mice lead to shortened limbs, snout and multiple tissue defects

**Fig. 1.**
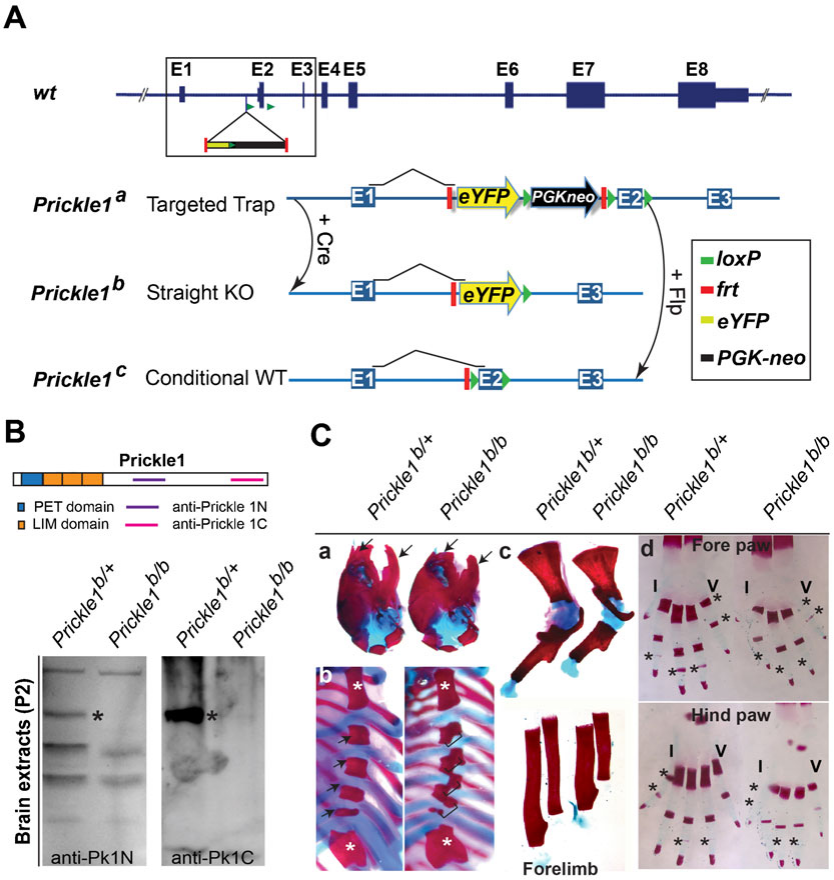
Generation of *Prickle1* mutant alleles, and skeletal defects in *Prickle1* null mutants. (A) Genomic structure and targeting strategy (box) at the *Prickle1* gene locus is shown at the top (*wt*). *Prickle1^a^* is a gene-trap allele with an inserted *eYFP* reporter (yellow arrow), *neo* selection cassette (black arrow), *Frt*s (red bars) and *loxP*s (green arrowheads) as shown in the graph. *Prickle1^b^* is a null allele (straight KO) derived from *Prickle1^a^*, having undergone cre-mediated excision of exon 2 with starting ATG, and the *Pgk-neo* cassette in the germline. *Prickle1^c^* is a conditional allele generated by Flp-mediated excision of the *eYFP* and *Pgk-neo* cassettes in *Prickle1^a^*, with two remaining *loxP*s flanking exon 2 that could be further excised by a tissue-specific Cre. (B) Western blots probed with custom-made Pk1 antibodies revealed elimination of Pk1 protein expression in the null (*Pk1^b/b^*) brain tissue collected at postnatal day 2 (P2). * Denotes Pk1 protein band. (C) Skeletal structures revealed by alcian blue and alizarin red staining. a, Craniofacial and skull skeleton: arrows indicate premaxillary and dentry bones. b , Rib cages: Brackets indicate sternebrae; top asterisks indicate sternal manubrium, bottom asterisks indicate xiphisternum. c, formlimb long bones: top panels, scapula and humerus; bottom panels, radius and ulna. d and e, Digit phalange bones of fore paw and hind paw, respectively. ‘I’, digit I; ‘V’, digit V. Asterisks indicate phalanges bones with absent or delayed ossification centers.

We generated three mutant *Prickle1* alleles in mice by targeting exon 2 with the starting ATG: a gene-trap allele (*Prickle1^a^*), a null allele (*Prickle1^b^*), and a conditional allele (*Prickle1^c^*) (Fig. 1A). The gene-trap allele was predicted to be a hypomorph and produce a trace amount of protein, whereas *Prickle1^b^* and Cre-excised *Prickle1^c^* alleles would be null systemically or in targeted tissues. We first verified that Prickle1 protein was abolished in *Prickle1^b/b^* mutant by immunoblot analysis using two independent antibodies against different domains of Prickle1 (Fig. 1B). In contrast to the reported *Prickle1* null mutant that exhibited embryonic lethality prior to gastrulation (Tao et al., 2009), the null mutants (*Prickle1^b/b^*) we produced survived until postnatal day 2 (P2). The *Prickle1^c/c^* conditional deletion generated by a broadly expressed *Sox-cre*, which effectively represented a null genotype in most tissues, survived for about 2 days longer. The morphological defects of *Prickle1^b/b^* mutant mice were seen as early as embryonic day (E) 15.5 and become more evident at P0 with shortened limbs and snout (supplementary material Fig. S1A,B). A close view of fore- and hind- paws showed blunted digit tips in the mutants (supplementary material Fig. S1C,D). Although the mutant mice are generally slightly smaller at P0, shortening of limb and snout are more pronounced than shortening of the body axis (supplementary material Fig. S1E,F). Additional phenotypes observed at P0 included open eyelid (supplementary material Fig. S2A), bifid and truncated tongue (supplementary material Fig. S2B), cleft palate (supplementary material Fig. S2C, >17 out of 31), curly tail (supplementary material Fig. S2D) and hair orientation defect (supplementary material Fig. S2E,F). Similar phenotypes were seen in Sox2-Cre conditional mutants (supplementary material Fig. S2E,G). Skeletal staining with alcian blue and alizarin red (Fig. 1Ca–e) revealed shortened nasal, premaxillary and dentary bones (Fig. 1Ca), misaligned sternebrae (Fig. 1Cb, 19 out of 31), shorter but thicker long bones (Fig. 1Cc, upper: scapula and humerus; lower: radius and ulna) and absent or delayed ossification centers of phalangeal bones (Fig. 1Cd). Extensive internal organ defects were observed some of which will be described below (and data not shown).

### Cardiac outflow tract defects and disorganized myocardial fibers in the *Prickle1* mutants

**Fig. 2.**
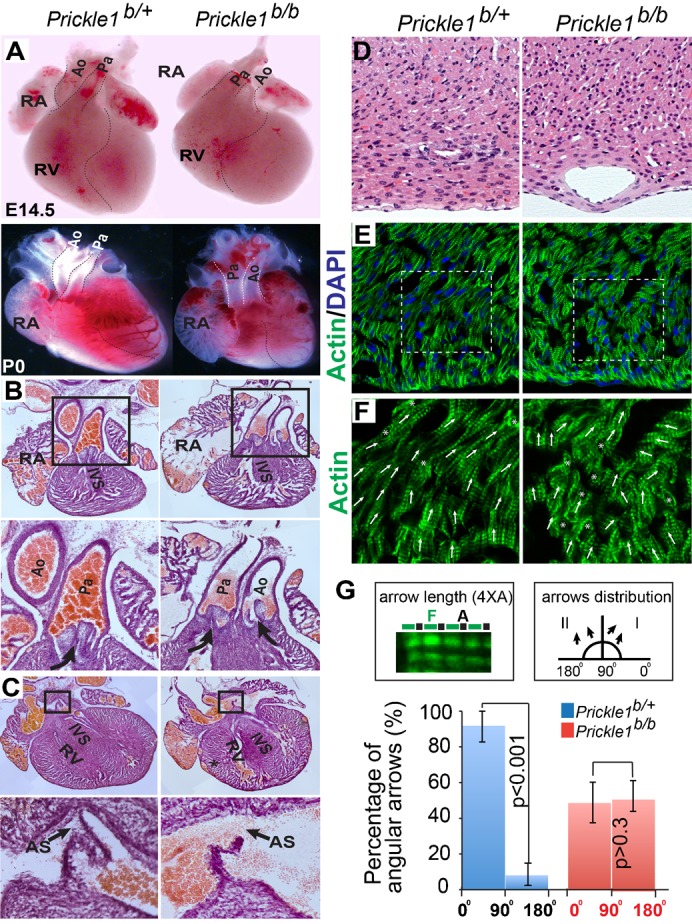
Transposition of great arteries, opened atrial septum and disorganized cardiac myofibers. (A) Embryonic heart from E14.5 (upper panels) shows transposition of pulmonary (Pa) and aorta (Ao) arteries with shortened apex-base axis. Lower panels are P0 heart. RA, right atrium; RV, right ventricle. Dotted lines mark the boundaries of great vessels and ventricle separations. (B) H&E stained sections through P0 heart showing transposed Pa and Ao in the mutant heart. The normal position of Ao is right above the Pa (left panels), while Ao is juxtaposed with Pa in the mutant heart. Zoomed-in boxed areas are shown in lower panels. Curved arrows point the blood flow paths from ventricle to the great arteries. (C) The atrial septum is left open in the mutants. Zoomed-in boxed areas are shown in lower panels. The arrow indicates blood passing through both atrial chambers in the mutant. (D) H&E stained plastic sections of P0 heart. The control myocytes nuclei are elongated whereas the mutant nuclei are rounder. (E) Phalloidin-stained myofibrils. The mutant shows twisted and shortened weaving pattern. Boxed areas are zoomed in F and quantified in G. (F) Orientations of myofibrils indicated by angular arrows arbitrarily drawn along Phalloidin-stained thin actin. Each arrow marks at least 4 continuous A-bands of myofibrils (see G, top left box). Asterisks indicate fibers that travel vertical to the image plane. (G) Quantification of myofibrils' orientations in two dimensions referring to epicardium surface, on which rays from any given points are defined as 0 and 180 degree angles, respectively, in opposite directions (right versus left). All angles are forced into quadrant I–II (0–180°) by subtracting 180 if they happen to fall into quadrant III–VI (180–360°). Top left box is a close-up view of a myofibril segment comprises at least 4 A-bands for each angular arrow. Top right box is an angle-plotting scheme in 90° bins; arrows distribution from 0° to 180° within each bin is presented as percentage of total. Error bars are standard deviations. Three control or mutant animals were used and three tissue sections from each animal were collected for quantification. A total of 108 and 111angular arrows from control and mutant tissue sections were analyzed, respectively.

Malformation of the heart, a PCP target organ, causes postnatal death in many instances. In *Prickle1* mutants, heart chambers and outflow tracts were developed at E14.5 (Fig. 2A). The aorta (Ao) and pulmonary arteries (Pa), however, were transposed in more than half of the mutant embryos (19 out of 31). Similar phenotypes were observed in newborn (P0) mutant pups, with significantly enlarged right atrium (Fig. 2A, lower panels; 2B, upper panels) and juxtaposed Ao and Pa (Fig. 2B). The atrial septum was also open (Fig. 2C). At the cellular level, H&E staining showed round mutant cardiomyocyte nuclei in contrast to the spindle-shaped nuclei of the control, indicating the loss of polarity (Fig. 2D). Actin filament in myofibrils were disorganized and misaligned (Fig. 2E,F). A significant increase of randomness in fiber orientation was also noticeable in the mutants (Fig. 2F,G), a novel feature that has not been reported in other PCP mutants.

### Distorted renal tubules and cell misarrangement in the mutant kidney

**Fig. 3.**
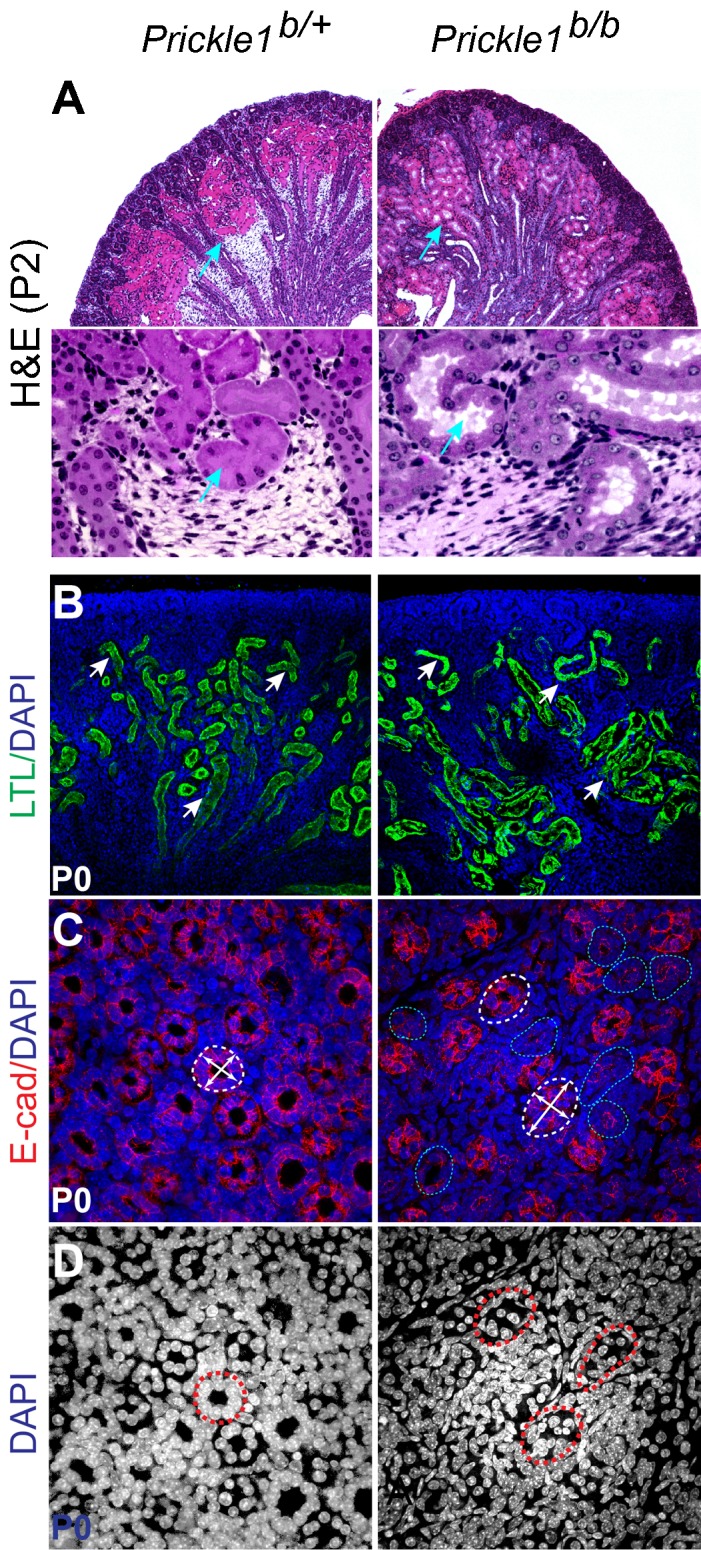
Distorted renal tubule shape in the *Prickle1* mutant kidney. (A) H&E stained kidney plastic sections showing dilated tubules in the renal cortex (arrows). Lower panels are high magnification images from roughly the same areas from the respective top panels. (B) *Lotus tetragonolobus lectin* (LTL) stained proximal convoluted tubules (PCT) (green). Loss of the uniform PCT shape is obvious in the mutant kidney in comparison with the wild type (arrows). (C) Cross sections of E-cadherin stained collecting ducts (CD) in the renal papilla. The control CDs showed well-formed circular shape, while majority of mutant CDs was irregular and shows elliptical shapes (dashed circles). Double crossing arrows indicate longest and shortest axes of tubules wall on cross sections. (D) DAPI staining alone from the same section in C can barely identify tubule structure in the mutant.

**Fig. 4.**
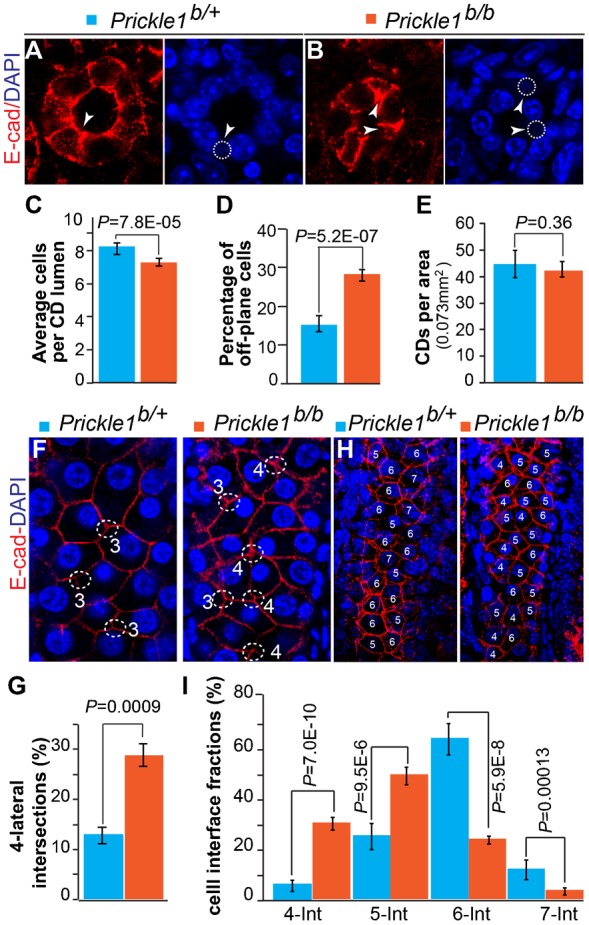
Impaired cell connection/arrangement in the mutant collecting ducts and renal tubules. (A–E) Quantification of CD wall composition. E-cadherin and DAPI staining were used to identify CD wall on cross sections (A,B). The diameters of CD were measured as number of cells. Cells out of an imaged plane (off-plane cells, arrows in A and B) of a CD wall but still can be identified by DAPI and E-cadherin staining (dashed circles) were counted as part of the tubule wall (arrows). Each imaged plane is composed of 7×1 µm-projected stacks. There is a slightly decrease in average CD diameter in the mutant (C). (D) Significant increase of off-plane cells in mutant CD walls. (E) No significant changes in the mutant CD area density compared to the control. (F) Ascending limb of the Henle's loop stained by E-cadherin that marks the cell junctions. Most of cell intersections in the control possess 3 laterals (3-lateral intersections, dotted circle), while many of them possess 4 laterals (4-lateral intersections) in the mutant. (G) Quantification of 4-lateral intersections in the control and the mutant tubules. (H) Representative images for quantification of the interfaces of the epithelial cells in descending Henle's loop. Numbers indicate the interfaces of a cell. The interfaces of a cell (-int) are defined by the number of cells it directly contacts with, which was visualized by both E-cadherin and DAPI staining. (I) Quantification of the cell interfaces. A total 331 wild type and 416 mutant cells derived from three animals of each genotype were analyzed. T-test was used to determine P-values. A significant increase of 4- and 5-int cells and a decrease of 6- and 7-int cells were observed in the mutants.

We next examined the kidney, another PCP target organ in which branching and tubule diameter are affected in *Vangl2* (Yates et al., 2010) and Wnt9b (Karner et al., 2009) mutant mice, respectively. Cystic kidney has also been observed in Robinow syndrome patients with mutations in Ror2 (Bacino, 1993), a receptor for Wnt5a (Ho et al., 2012). Kidney size in the *Prickle1* mutants was comparable to that of the control. There were frequently blood spots on the surface (supplementary material Fig. S3A, 24 out of 30 pulps). The development of renal tubules was largely normal judged by calbindin-D28K labeled ureteric bud (UB) and E-cadherin labeling (supplementary material Fig. S3B). Overt cystic kidneys were observed at birth albeit at very low penetrance (<5%, 3 out of 61) (supplementary material Fig. S3C). H&E staining showed dilated renal tubules in the mutants (Fig. 3A). *Lotus tetragonolobus lectin* (LTL)-labeled proximal convoluted tubules (PCT) appeared to be distorted, with narrowing of PCT in the outer renal cortex but widening of the inner renal cortex (Fig. 3B). The collecting ducts (CD) of the renal papilla were barely discernable by DAPI staining alone (compare Fig. 3C,D). E-cadherin staining showed irregular elliptical shape of the mutant collecting ducts (Fig. 3C). With simple quantification (Fig. 4A–D), we detected a significant increase in displaced/disarranged cells of the mutant lumen as revealed by E-cadherin and DAPI staining (off plane cells in Fig. 4A,B, arrowheads). No change was obvious in the collecting duct density per unit area (Fig. 4E). Quantification of cell-cell connections from a longitudinal view of collecting ducts (supplementary material Fig. S4A) revealed increased 4-lateral intersections of the mutants (supplementary material Fig. S4B).

To further investigate whether the kidney tubules were generally disorganized, we quantified cell alignment defects in the ascending Henle's loop, which has different origin from the collecting ducts during nephron formation. The thick ascending limb of the Henle's loop was easily identified by E-cadherin staining exhibiting a large plane of cuboidal epithelial cells (Fig. 4F). Cell-cell intersection pattern was significantly altered in the mutant with increased 4-lateral intersections in contrast to predominantly 3-lateral intersections in the control (Fig. 4F,G). Most cuboidal epithelial cells in the control were convex hexagons, but appeared irregular in the mutants with many more quadrilaterals and pentagons (Fig. 4H,I). The altered intersection mode and shape of the mutant epithelial cells were also accompanied by aberrant cell alignment. To quantify this feature, we first define a shortest compound line path between two adjacent columns of cells along the vertical direction of a tubule (supplementary material Fig. S4C,D). We then performed vectorial additions for laterals from each cell vertex on the compound line (supplementary material Fig. S4D) and acquired collections of new vectors. In a plotted polar coordinate (supplementary material Fig. S4E), significantly increased vector variations were observed in the mutants by paired t-test analysis (supplementary material Fig. S4E, P = 2.4E-07, Length: P = 2.02E-06). The vectorial variations in the mutants may indicate a decreased tubule stability and vulnerability to distortion.

### Neural tube closure and axon outgrowth defect

**Fig. 5.**
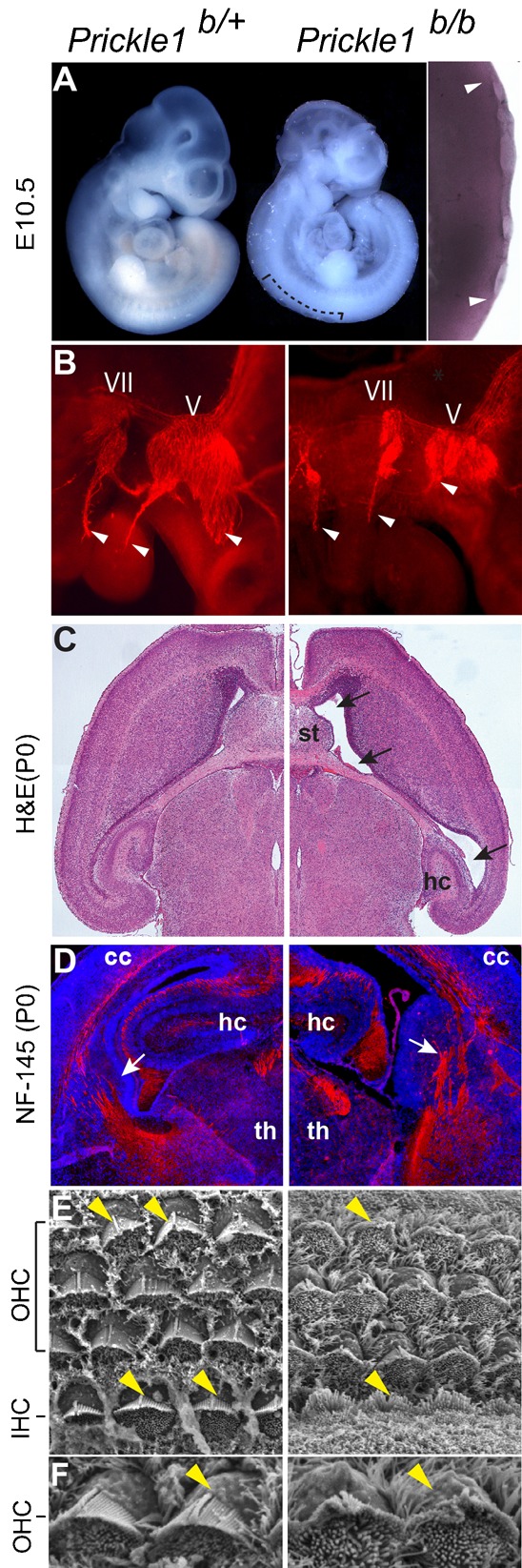
The nervous system defects in *Pk1* mutants. (A) E10.5 wild type and mutant embryos. The mutant embryo shows gross normal structure with retarded neural tube closure (the curved bracket, and zoomed in the side panel). Arrowheads indicate the boundary of open neural tube, asterisks indicate the opened areas. (B) Neurofilament stained E10.5 trigeminal nerves showing that V and VII brachial nerves innervations are stalled in the mutant (arrowheads). (C) H&E stained plastic section showing enlarged lateral ventricle (arrows), malformed striatum (st) and hippocampus (hc). (D) Coronal sections of P0 brains stained with anti-neurofilament showed that axonal tract (arrows) is impaired in the mutant. cc, cerebral cortex; th, thalamus. (E,F) Scanning electron microscopy (SEM) of the inner ear hair cells. (E) In the control, one row inner hair cells (IHC) and three rows of outer hair cells (OHC) roughly reside in same plane. However, the third row of the OHCs and the IHCs of the mutant tilted off the plane (arrowheads, right panel). (F) Larger magnification of the second row of OHCs showing disorganized actin bundles in the mutant hair cells (arrowheads).

Open neural tube is a typical PCP defect identified in Fz, Dvl, Celsr and Vangl mutants (Curtin et al., 2003; Etheridge et al., 2008; Murdoch et al., 2001; Wang et al., 2006). In *Prickle1* mutants, the neural tube was largely normal with a slight delay in closure at E10.5 (Fig. 5A, 8 out of 15). We observed brain and axon outgrowth defect as early as E10.5 (3 out of 4). Neural filament staining indicated the failure of innervation of trigeminal (V) and facial (VII) nerves (Fig. 5B). At P0, H&E staining showed enlarged lateral ventricles and misshaped hippocampus (Fig. 5C). The thalamic-cortical axonal tract was also disorganized in the *Prickle1* mutants (Fig. 5D, 3 out of 5). However, scanning electron microscopy (SEM) did not reveal stereocilia rotation defects in the inner ear hair cells that are typically observed in other PCP mutants. Rather, the stereociliary actin bundles of the *Prickle1* mutant hair cells were shorter and splayed at the tip (Fig. 5E,F, 3 out of 3), consistent with the actin filament defects identified in the heart.

### Cell polarity machinery and actin assembly defects in the *Prickle1* mutant renal tubules

**Fig. 6.**
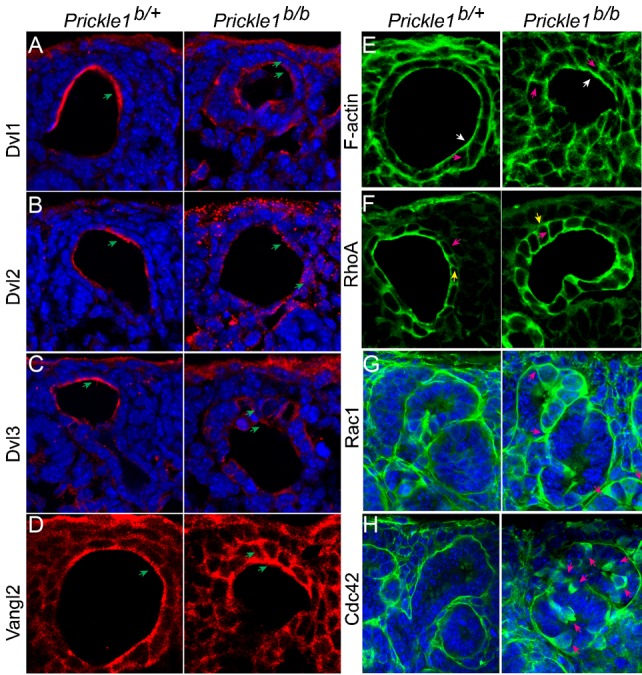
Mislocalization of Dvl, Vangl and small GTPases in the mutant ureteric bud. (A–D) Apically localized Dvl1∼3 and Vangl2 became mislocalized along the mutant ureteric bud wall. Arrows indicate apical localized and mislocalized proteins in the wild type and the mutant, respectively. (E) Mislocalization of F-actin in the ureteric bud (arrows). Enhanced lateral labeling of actin (red arrows) in the mutant in contrast to the predominant apical localization in the wild type. (F) Enhanced basal (yellow arrows) and lateral (red arrows) RhoA staining in the mutant in comparison with the predominant apical staining in wild type cells. (G,H) Ectopic elevated Rac1 (G) and Cdc42 (H) labeling in the mutant ureteric bud (asterisks, right panels).

Since PCP components were distributed asymmetrically, we asked whether the localization of other PCP proteins, such as Dvl and Vangl, was perturbed in the *Prickle1* mutants. Dvl1-3 and Vangl2 are concentrated at the apical domain of the control ureteric bud cells (Fig. 6A–D, left panels), yet a significant fraction of these was mislocalized to the lateral and basal aspects of the epithelia in the mutants (Fig. 6A–D, right panels). The results are consistent with an earlier report of disrupted apicobasal polarity in *Prickle1* mutant embryonic epiblasts (Tao et al., 2009). Actin assembly is a downstream event of PCP, which involves small GTPases (Schlessinger et al., 2009). We found that, rather than apical enrichment, the actin distribution was shifted more laterally in the mutant ureteric buds (Fig. 6E). Consistent with this observation, RhoA also decreased its apical enrichment (Fig. 6F; supplementary material Fig. S5A). Rac1 and Cdc42 are important for lamellilepodia and filopodia formation, respectively, in many types of cultured cells. Interestingly, they are ectopically accumulated in many cells of the mutant ureteric bud and other types of tubules (Fig. 6G,H and supplementary material Fig. S5B,C arrows). Their misexpression/deposition could indicate significantly increased variations in cell motility and/or local rearrangement, either of which could underlie the distorted tubular shapes seen in the mutants.

### Disruption of asymmetrical distribution of core PCP proteins in the mutant chondrocytes

**Fig. 7.**
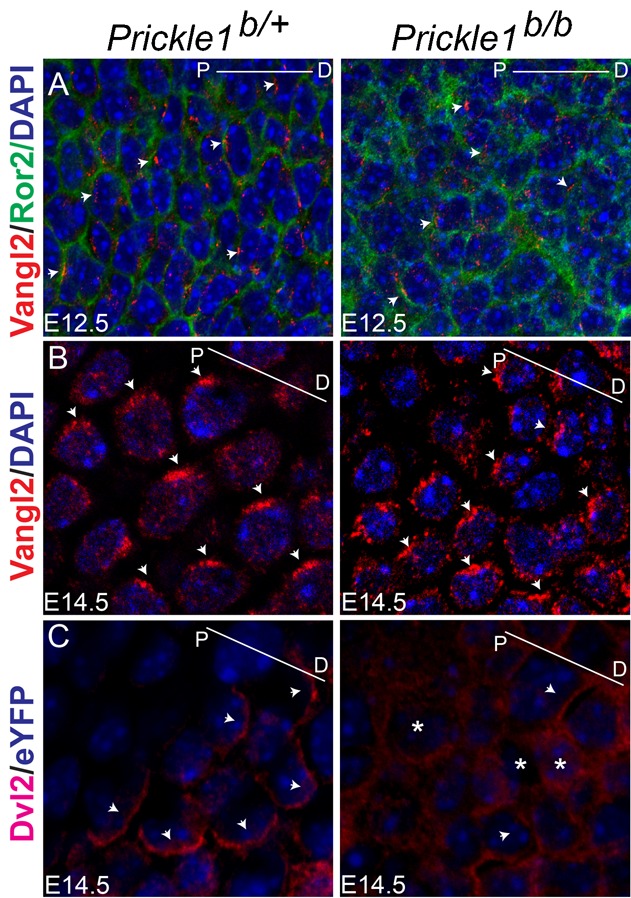
Vangl2 and Dvl2 localization in developing digit chondrocytes. (A) Zoomed-in area from supplementary material Fig. S6A. Polarization of Vangl2 staining (red) along proximal-distal axis in digit chondrocytes at E12.5. Note the poor polarization of Vangl2 in the mutant compared with that in the wild type chondrocytes. Ror2 is not polarized (green). (B) Zoomed-in area from supplementary material Fig. S6B. Polarized Vangl2 (red) surrounding the ossification center in both wild type and the mutant chondrocytes. Note more diffused localization of Vangl2 in the mutant chondrocytes. (C) Zoomed-in area from supplementary material Fig. S6C. Chondrocytes with polarized Dvl2 (red) at the digit tip at E14.5 (left panel) and loss of Dvl2 polarization in the mutant chondrocytes (asterisks, right panel). Lines in each panel indicate proximal-distal axes.

To understand the cause of limb outgrowth defects in the mutants, we examined two polarity proteins, Vangl2 and Dvl2, which were reported to mediate Wnt5a signaling crucial for limb outgrowth (Gao et al., 2011; Ho et al., 2012). Vangl2 localizes asymmetrically in chondrocytes at E12.5 (Gao et al., 2011). We found a similar, proximally enriched pattern of Vangl2 in the control chondrocytes but this asymmetry was diminished in the mutant (Fig. 7A; supplementary material Fig. S6A and Fig. S7A). Asymmetrical distribution of Vangl2 protein was also observed at E14.5 in the circumferential areas of the bone ossification center of the control, but the localization pattern was broadened in the mutants (Fig. 7B; supplementary material Fig. S6B), and mutant cells with polarized Vangl2 were reduced (supplementary material Fig. S7B). Further analysis demonstrated a reduction of alignment of the mutant cells with Vangl2 staining (supplementary material Fig. S7C). Although Vangl2 localization was reported to polarize along proximal-distal axis in general, it appeared to polarize radially surrounding the ossification center (supplementary material Fig. S6B). Dvl2 polarization was not detected at E12.5 in the control digit chondrocytes (data not shown) but emerged at E14.5 appearing as a distally enriched pattern and the opposite of Vangl2 (Fig. 7C and supplementary material Fig. S6C, left panel). This polarization was completely lost in the mutant (Fig. 7C and supplementary material Fig. S6C, right panel). These results indicated that similar to the kidney tubules, the cell polarity machinery in chondrocytes was also perturbed, which could explain the shortened limb phenotype.

### Disruption of Wnt5a signaling and Robinow symdrome-like features in *Prickle1* mutants

**Fig. 8.**
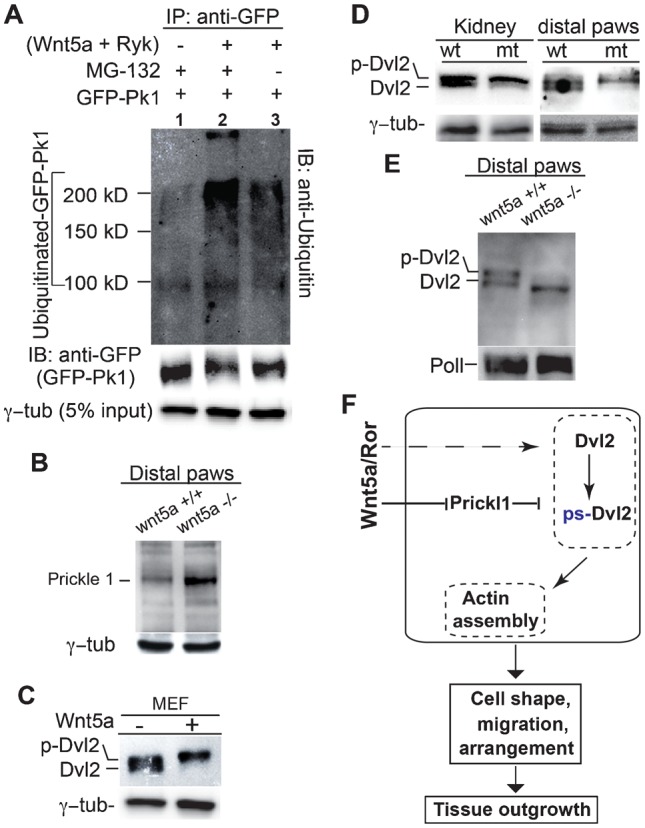
Disrupted Wnt5a signaling of Prickle1 mutant alleles. (A) Prickle1 proteasomal degradation on Wnt5a signaling. HEK293 cells were transfected with combinations of *GFP-Prickle1*, *Wnt5a* and *Ryk*. Anti-GFP antibody is used to immunoprecipitate Prickle1. Anti-ubiquitin antibody was used to detect ubiquitinated Prickle1 on an immunoblot. Increase in ubiquitinated Prickle1 is detected upon Wnt5a and Ryk transfection and treatment with MG-132 (lane2 and lane3 with lane1). The same blot was then probed with anti-GFP antibody to detect unmodified GFP-tagged Prickle1 (lower blot). The probable intact GFP-tagged Prickle1 protein roughly inversely correlates to the ubiquitination level (upper panel). (B) Upregulation of Prickle1 protein in *Wnt5a^−/−^* limb tissues of distal paws at E17.5. (C) Wnt5a stimulates phosphorylation of Dvl2 in MEFs. (D) Reduced unphosphorylated form (native form) of Dvl2 in the mutant kidney and limb tissues at P0, mimicking application of Wnt5a to cultured MEF cells. (E) Decreased dephosphorylated Dvl2 in *Wnt5a^−/−^* limb tissues of the distal paws at E17.5. Same Western blot was reprobed by anti-RNA polymerase II (PoII) for loading control. ps-Dvl2: Phosphorylation-shifted Dvl2. (F) A proposed role of Prickle1 in regulation of Wnt5a signaling- controlled cellular events. Prickle1 inhibits Dvl2 phosphorylation, which is critical for localized actin assembly and asymmetrical cellular localization of Dvl2. Aberrant Dvl2 phosphorylation would lead to defects in cell shape, cell migration and arrangement during tissue morphogenesis. Wnt5a signaling adjust status of Dvl2 phosphorylation partially through degradation of Prickle1.

To explore the relationship between Prickle1 and Wnt5a signaling, we first turned to *in vitro* assays in cultured HEK293 cells. We found that ubiquitination of a GFP-tagged Prickle1 was enhanced by Wnt5a and Ryk in presence of MG132, a proteasome inhibitor (Fig. 8A, compare lane2 with lane1). As expected, accumulation of ubiquitinated Prickle1 was less significant without MG132 treatment (Fig. 8A, compare lanes 2 and 3), presumably due to its rapid proteasomal degradation. These results suggest a negative regulation of Prickle1 by Wnt5a signaling. Accordingly, increased *Prickle1* accumulation was observed in *Wnt5a* null mutant tissues (Fig. 8B). Wnt5a enhances Dvl2 phosphorylation in several cell lines and in embryonic fibroblasts as well (González-Sancho et al., 2004; Ho et al., 2012) (Fig. 8C). We therefore examined Dvl2 expression in *Prickle1* mutants. A disproportional increase of phosphorylated and shifted form of Dvl2 (ps-Dvl2) was detected in both mutant kidney and distal paw tissues at P0 (Fig. 8D), suggesting that loss of Prickle1 potentiates Wnt5a signaling. To further corroborate that Dvl2 phosphorylation regulated through Wnt5a-Prickle1 is a critical event during limb outgrowth, we examined Dvl2 phosphorylation status in *Wnt5a* mutant distal paws. A decreased Dvl2 phosphrylation was observed (Fig. 8E), consistent with the finding that Wnt5a signaling degrades Prickle1. Taken together (Fig. 8F), our data indicate that Prickle1 mediates Wnt5a signaling to modulate Dvl2 phosphorylation/activation; the latter regulates actin assembly, a process affecting multiple aspects of tissue morphogenesis including cell shape, migration and arrangement.

**Fig. 9.**
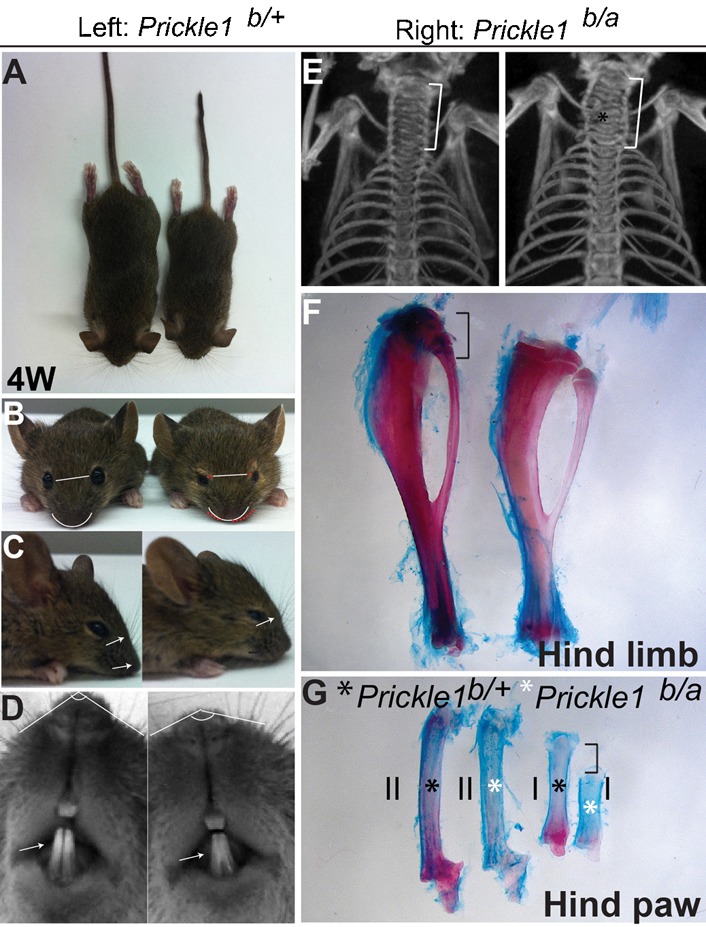
Robinow symdrome features presented in *Prickle1* mutant mice. (A) Top view of short statures of a *Prickle1^b/a^* hypomorphic mutant (*Prickle1^b/a^*, right) with a kinked tail. (B) Front view of control and hypomorphic mutant mice. Straight white lines indicate eye distance; curved white lines indicate nose curvature. The white line and curved line in the mutant are copied from the wild type, are therefore same in length and curvature. Red dotted and curved lines indicate expansion of eye distance and facial flatness in the mutant mouse, respectively. (C) Side view shows prominent forehead and less-pointed nose in the mutant (arrows). (D) Ventral view of the same control and mutant pair in c. Angles show pointiness of nose; arrows indicate incisors, which are not well separate in the mutant. (E) Computed tomography (CT) scan showing the distorted and incomplete cervical vertebrae defects in the mutants. (F) Hind limb long bones are shorten in the mutants. (G) Hind paw metatarsals of digit ‘I’ and ‘II’. Note digit ‘I’ is severely shorter, but ‘II’ is comparable.

Previous studies (Afzal et al., 2000a; Afzal et al., 2000b; Ali et al., 2007; Butler and Wadlington, 1987; Patton and Afzal, 2002) suggested that the tissue outgrowth and craniofacial defects in *Prickle1* mutants were reminiscent of human Robinow syndrome (RS). Since many RS patients have normal life expectancy, we examined whether *Prickle1* hypomorph mutants would survive to adulthood and display RS-like features. We generated mice with gene-trap (*Prickle1^a/a^*) alleles alone and with combined gene-trap and germline knockout (*Prickle1^a/b^*) alleles. Both *Prickle1^a/a^* and *Prickle1^a/b^* hypomorphs were born at the expected Mendelian ratio and survived to adulthood, but developed shorter stature (Fig. 9A) and craniofacial defects with wide spaced eyes, flat nose, short snout (Fig. 9B), prominent forehead (Fig. 9C) (30 out of 30), abnormal appearance of eyelid and eyelashes (supplementary material Fig. S8A–D, 15 out of 15), and fused mandibular incisors (Fig. 9D, 14 out of 30). Computed tomography (CT) scans detected incomplete and distorted vertebrae formation in the mutant spinal cord (Fig. 9E, 3 out of 3). Significantly shortened long bones (Fig. 9F, Femurs) and digital bones were consistently observed (Fig. 9G, metatarsals, 3 out of 3). Thus *Prickle1* hypomorphs recapitulate most phenotypic features of human RS patients (Butler and Wadlington, 1987; Patton and Afzal, 2002). Our findings uncover an unexpected aspect of RS pathophysiology and suggest that loss-of-function mutations in human *PRICKLE1* might also be a cause of this condition.

## DISCUSSION

Organization of similar and/or different cell types throughout a large field is crucial for systemic functions. PCP is among the best-known signaling pathway managing large-scale cell organization. In mammals, disruption of different homologues of insect core-PCP proteins suggest variable functions of PCP components in distinct cellular processes during embryonic development. For example, disruption of *Frizzleds* (Wang et al., 2006), *Dvls* (Etheridge et al., 2008), *Celsr1*(Curtin et al., 2003) and *Vangl2* (Kibar et al., 2001) exhibit some phenotypic commonalities including open neural tube and misoriented inner ear stereociliary bundles, which are considered classic PCP phenotypes. Disruption of *Diego*/*Inversin* lead to left-right asymmetry defects without significant classic PCP phenotypes (Morgan et al., 1998; Simons et al., 2005; Watanabe et al., 2003).

Wnts are suggested to be functionally linked to the activity of core-PCP components regardless of whether these are part of or parallel to the core-PCP. Wnts have been demonstrated in flies and lower vertebrates as critical morphogens for the core PCP complex (Takeuchi et al., 2003; Veeman et al., 2003; Wu et al., 2013). However, disruption of *Wnt5a*, a known PCP-related morphogen in mammals, gives rise to characteristic shortening of distal tissue axes (Gao et al., 2011; Ho et al., 2012; Qian et al., 2007), which is barely seen in other PCP mutants. Furthermore, mutations in either *Wnt5a* or its receptor *Ror2* in humans lead to Robinow syndrome, distinct from that of other PCP gene mutations (Kibar et al., 2007; Murdoch et al., 2001; Wang et al., 2011). In the present study, we show that null and hypomorph *Prickle1* mutations in mice manifest clinical features of human RS caused by mutations of Wnt5a pathway. Wnt5a regulates Prickle1 degradation to signal Dvl2 phosphorylation further suggest that Prickle1, unlike other identified core PCP component, is dedicated to Wnt pathway, which is particularly important for dynamic cell arrangement and migration during tissue outgrowth. The phenotypic similarity and its role in mediating Wnt5a/Ror2 signaling make *Prickle1* a likely candidate gene for Robinow syndrome.

We initially generated different *Prickle1* alleles in order to bypass early embryonic lethality reported previously in a *Prickle1* null mutant (Tao et al., 2009). Our goal was to study *Prickle1* late-stage PCP function, which cannot be addressed using conventional knockout mice. Surprisingly, the *Prickle1* null embryos in our colonies survived through gastrulation, and mutant mice were born at the expected Mendelian ratio. Our *Prickle1* gene targeting strategy was identical to that of the previous study (Tao et al., 2009). In both studies, exon2 carrying the protein translation start codon was deleted. This should result in a null allele, which we were able to confirm directly by showing absence of residual or aberrant proteins on immunoblots using both N and C-terminal Prickle1 antibodies. Furthermore, we replaced exon2 by an *eYFP* reporter carrying its own poly(A) signal, which should cause RNA transcription to stop and thus a further disruption to *Prickle1* gene expression. Therefore the stark discrepancy in mutant survival between the two studies cannot be attributed to a differential degree of gene ablation. Rather, this difference is most likely due to the difference in genetic background, which in our case is an equal mixture of C57Bl/6 and Sv/129 but a pure C57Bl/6J through backcrossing in the prior study.

The pleiotropic tissue outgrowth defects in *Prickle1* mutants indicate a disruption of PCP pathway that govern morphogenesis. The vectorial cell disarrangement in *Prickle1* mutant tissues including in kidney and heart demonstrates a role of Prickle1 in PCP function. However, PCP defects in *Prickle1* mutants appear to mechanistically differ from those in other PCP genes. Mislocalization of PCP proteins, small GTPases and F-actin are reminiscent of disrupted AB polarity, which is not usually seen in other PCP mutants. This part of our data is consistent with the suggested role of Prickle1 in embryonic epiblast AB polarity (Tao et al., 2009). These observations indicate that Prickle1 may also be involved in setting up apico-basal polarity.

Our study suggests that *Prickle1* is a major component of Wnt5a signaling. Several lines of evidence support this conclusion: i) Prickle1 is targeted to proteasomal degradation upon Wnt5a signaling; ii) Prickle1 is upregulated in *Wnt5a* null mutant limb tissue; and iii) Dvl2 phosphorylation, a hallmark of Wnt5a activation (González-Sancho et al., 2004; Ho et al., 2012), is altered in Prickle1 mutant tissues. Published studies demonstrating ubiquitination of Prickle1 by Smurf/Par6 complex upon Wnt5a signaling (Narimatsu et al., 2009) and Dvl2 stability regulated by Prickle1 (Carreira-Barbosa et al., 2003) provide additional support for notion that Prickle1 transduce Wnt5a signaling to downstream targets. A previous study demonstrated that Vangl2 is a phosphorylation target upon Wnt5a/Ror2 signaling, and Vangl2 polarization is lost upon disruption of Wnt5a signaling in chondrocytes (Gao et al., 2011). However, in Ror compound mutant MEF cells, Dvl rather than Vangl2 phosphorylation is found to be a critical event (Ho et al., 2012). Similarly, our data showed that Dvl2 phosphorylation is a downstream target of Prickle1-mediated Wnt5a signaling. We also found that Vangl2 polarization is partially retained in Prickle1 mutant chondrocytes, suggesting that Prickle1 and Dvl2 may act downstream of Vangl2.

Loss of Prickle1 leads to enhanced Wnt5a signaling in biochemical assays yet the mutant phenotypes mimic those of both gain- and loss-of-function mutants of Wnt5a (van Amerongen et al., 2012; Yamaguchi et al., 1999) (supplementary material Fig. S9). This paradox could be explained by the nature of polarity signaling. Namely, Wnt5a gradient confers a vectorial (directional) cue that drives cell behavior. Both absence of Wnt5a or excess Wnt5a that saturates signaling around the entire cell will lead to loss of the vectorial information (supplementary material Fig. S10). Loss of Prickle1 disrupts Wnt5a signaling to downstream targets so that cells cannot interpret or respond to the vetorial information. Hence all three genetic alterations lead to a similar defect in tissue morphogenesis.

## MATERIALS AND METHODS

### *Generation of Prickle1 mutant alle*les

All procedures involving the use of mice were approved by National Eye Institute Animal Care and Use Committee (ACUC). *Prickle1* gene-trap mutant strain was generated as described previously (Liu et al., 2013). The knockout construct was designed as a gene-trap allele first and was brought into straight and conditional knockout alleles upon excisions by Cre and Flp recombinase, respectively. Briefly, the following elements were engineered into *Prickle1* locus in order: *Frt*, *En2* splicing acceptor, *eYFP*, polyadenylation signal (poly (A)), *loxP*, *PGK-neo*, *Frt*, and *loxP* right before *Prickle1* exon 2, and a third *loxP* after Exon2. This allele allows *eYFP* reporter expression under the control of endogenous *Prickle1* promoter, and was designated as *Prickle1^a^* (Fig. 1A). A complete Cre and Flp recombination will convert *Prickle1^a^* to *Prickle1^b^* and *Prickle1^c^* alleles, respectively (Fig. 1A). Southern analysis and genotyping were conducted as described previously (Liu et al., 2013).

*Wnt5a* knockout mouse strain was obtained from JAX laboratory, and maintained by standard protocol that complies with ACUC regulations at NEI.

### Histology, immunohistochemistry and imaging

Mouse skeletal visualization entirely followed protocol by Ovchinnikov (Ovchinnikov, 2009). Images were taken with dissection Zeiss microscope equipped with Axiovision digital camera. Tissues were dissected and fixed with 4% PFA and subjected to sectioning at 15 um for frozen sections, or 50 um for vibratome sections. H&E staining was performed on fixed frozen sections (*FD Neurotechnologies*, Inc, PS103-1, PS-104-1), and on methacrylate plastic sections generated at NEI Histology core. Immunohistochemistry were performed as previously described (Liu et al., 2012). Skin flat mount was prepared exactly following the description by Wang et al. (Wang et al., 2010). Briefly, skin was dissected and cleaned away of fat tissues, fixed with 4% PFA, and cleared with benzyl benzoate:benzyl alcohol (BBBA), imaged using a Zeiss dissection microscope (Liu et al., 2008). Antibodies and fluorescent dyes were used in this study are as follows: Phalloidin-Alexa 488, (Invitrogen, A12379); *Lotus tetragonolobus lectin*, (Vector Laboratories, FL-1321); rat anti-E-cadherin, (Sigma, U3254); mouse anti-calbindin D28K, (Sigma, C9848, CB-955); mouse anti-α–acetylated tubulin, (Sigma, T6793); mouse anti-Dvl1, (3F12, Santa Cruz Sc-8025); mouse anti-Dvl3, (4D3, Santa Cruz : SC8027); mouse anti-Dvl2 (Santa Cruz, sc-8026); mouse anti-Ror2 (DSHB), rabbit anti-Vangl2, (gift from Dr. Matthew Kelley at NIDCD, NIH); mouse anti-RhoA, (Sigma, SAB1400017; Cytoskeleton, Inc. Cat. ARH03 ); β-catenin (610154, BD transduction laboratories); rabbit anti-NF-145, (Chemicon, AB1987); mouse anti-Cdc42, (Cytoskeleton, Inc, Cat # ACD03); mouse anti-Rac1, and (Cytoskeleton, Inc, Cat # ARC03). Fluorescent images were collected using Leica SP5 and Olympus FV1000 confocal microscope at NNRL and NEI imaging core. Fluorescent intensity measurement was conducted using NIH ImageJ software.

### Quantification of cardiac myofibrils' orientation, renal tubules' cell arrangement, cell alignment and interfaces

The cardiac myofibrils orientation is defined by a group of arrows arbitrarily drawn along Phalloidin stained actin. Each arrow marks at least 4 continuous A-bands (Fig. 2H). Angles of arrows are measured referring to epicardium surface, on which rays from any given points are defined as 0 and 180 degree angles respective to opposite directions (right versus left). All angles are forced into quadrant I–II (0–180°) by subtracting 180° if they happen to fall into quadrant III–VI (180–360°). Arrows are binned into 0°–90° and 91°–180°, and presented as percentage of total in graph. About 35 angular arrows/section were drawn to illustrate myofibrils' orientations. Three tissue sections from each animal were used for quantification. Three animals for each genotype were used for analysis. Total 108 and 111 angular arrows from respective control and the mutant tissue sections were measured.

Thickness of a collecting duct (CD) wall was quantified as cell number. E-cadherin and DAPI staining was used to identify cells making up the CD wall on cross sections. Each imaged plane is composed of 7×1 µm-projected stacks. Cells out of an imaged plane are named off-plane cells. Three tissue sections from three animals of each genotype were used for analysis. About 90 tubules were counted for each genotype. Student *t*-test was used to detect the P value for all groups of data.

Cell arrangement was quantified by examination of cell intersection pattern in the thick ascending limb of the Henle's loop. Depending on number of cell laterals (junctions) intersect, cell arrangement is defined as 3-lateral and 4-lateral patterns. Cell alignment was quantified as a transformation of lateral vectorial lengths of two adjacent columns of cells. A shortest compound line path between two adjacent columns of cells along the vertical direction of a tubule was drawn (supplementary material Fig. S4C). Vectorial additions were then performed for laterals from each cell vertex on the compound line using parallelogram method to create collections of new vectors (supplementary material Fig. S4D). These vectors were plotted in a polar coordinate (supplementary material Fig. S4E). The orientations and lengths of the vectors variations were subjected to paired t-test to detect statistical differences. 68 vectors acquired roughly equally from three animals of either wild type or the mutant Henle's loop were analyzed and plotted.

Cell interfaces of a Henle's loop cell were quantified by counting cells directly contacting that cell. Because the shapes of many mutant cells are not regular, we therefore named the cells by their interfaces (int). Three tissue sections from each animal were used for quantification. Three animals for each genotype were used for analysis. Total 331 wild type and 416 mutant cells were cataloged.

### Quantification of alignment of Vangl2 staining

Cells with positive Vangl2 staining were counted. Fractions of polarized Vangl2 cells were presented as percentage. A set of three sections derived from either wild type or mutant embryos were used for analysis. P-values were computed by T-test function. The aligned cell groups were defined only if three center points of Vangl2 staining of adjacent cells make an angle equal or large than 165 degree (arbitrarily defined), for example angles a, b and c (supplementary material Fig. S7). By this definition, the aligned cells do not deviate more than 15 degree from each other in terms of stained Vangl2 orientations. Three wild type and three mutant embryos were analyzed, each of which 24 cell groups were analyzed. A t-test was conducted using the Excel program to compute p values.

### Tissue culture and transfection

Culture medium used for MEFs and HEK293 cells is based on DMED/F12 (Invitrogen, *Cat.* 12660-012) supplemented with or without 10% FBS and penicillin/streptomycin (Invitrogen, Cat. 15070-063). Cell transfection was performed in 6-well dishes with total amount 2 ug DNA/well using Lipofectamine 2000 (Invitrogen, Cat. 11668-019) for different combinations of GFP-Pk1, Wnt5a and Ryk plasmids. Biological triplicates and technical replicates were made for each transfection. 36 hr after transfection, cells were treated with 100 nM DMSO or MG-132 for 20 hr. Cells were then harvested for preparation of protein extracts.

For culture primary mouse embryonic fibroblasts (MEFs), E13.5 embryos were dissected out from the uterus of pregnant females in HBSS (Invitrogen, 14025-076), internal organs and head were removed, remained tissues were chopped into pieces using a razor blade, digested with 2.5% trypsin at room temperature for 15 min with soft taps every 5 min, pipetted through 1 ml pipette tip for multiple times, added DMEM/F12 (Invitrogen, Cat. 12660-012) medium with supplement of 10% FBS and penicillin/streptomycin (Invitrogen, Cat. 15070-063), and filtered through 70 µm cell mesh strainer (Biologixresearch, 15-1070). Cells were collected by centrifuge at 1000× g, plated into 10 cm culture dish, and used at passage 2.

### Immunoprecipatation and immunoblotting

Protein extracts from tissue or transfected HEK293 cells were prepared with RAPI or 2×SDS sample buffers according to the standard protocols. Protein A Dynabeads (Invitrogen, Cat.# 1000D) were used for pulling down anti-GFP antibody (Rabbit anti-GFP , Torrey Pines Biolabs, Inc, TP401) associated Prickle1 protein complex at 4C overnight. Proteins were dissociated from the beads by heating in 2×SDS sample buffer at 98C for 5 min, then run a western blot and probed with anti-Ubiquitin antibody and anti-GFP antibody.

Custom-made antibodies for Prickle1 were generated in our laboratory by immunizing rabbits with bacterially expressed recombinant proteins corresponding to aa 344–464 (Pk1C) and aa 711–849 (Pk1N), respectively. Antisera were affinity purified and were used at 1:1000 dilutions for Western blots. Mouse anti-γ-tubulin (Sigma, T-6557) was used at 1:3000; Rabbit anti-Wnt5a was used at 1:1000 (Abcam, ab72583); RhoA, Cdc42 and Rac1 (Cytoskeleton, Inc) were used at 1:1000; rabbit anti-Dvl2 (Chemicon, AB5972; Cell Signaling, Cat# 3216) and rabbit anti-GFP were used at 1:2000; mouse anti-Ubiquitin antibody (ab7254) was used at 1:1000.

### SEM ultrastructure analysis of the inner ear cells in cochlea

Samples were prepared for SEM as described (May-Simera and Kelley, 2012). Dissected cochlea sensory epithelia were fixed in EM fix (2.5% glutaraldehyde, 4% PFA, 10 mM CaCl_2_ in 0.1M Hepes) for 2 hr at room temperature. After coating with repeated washes of 1% OsO_4_ and 1% tannic acid, samples were dehydrated through an ethanol series, critical point-dried, and imaged on a S-4800 (Hitachi) field emission scanning electron microscope.

### X-ray micro computed tomography (micro-CT imaging)

X-ray micro computed tomography (micro-CT imaging) is performed in the Mouse Imaging Facility. The mouse is placed under general anesthesia in an induction chamber with 3–5% isoflurane delivered by a gas mixture of oxygen, medical air and nitrogen. Once the animal is unconscious, anesthesia is maintained with 1–2% isoflurane administered via nosecone. Sterile ophthalmic ointment is applied to the corneas to prevent desiccation under anesthesia. The animal is placed on the imaging platform secured into the necessary imaging position using transpore and autoclave tape. Depth of anesthesia is monitored by direct visualization of the animal respiratory rate. Body temperature is maintained by the imaging systems warm air blower. Anesthetic gasses are scavenged in a centralized vacuum system approved by the Division of Safety. Total imaging time is approximately 5–30 minutes for a 180 or 360 degree scan.

## Supplementary Material

Supplementary Material

## References

[b1] Afzal A. R. Rajab A. Fenske C. Crosby A. Lahiri N. Ternes-Pereira E. Murday V. A. Houlston R. Patton M. A. Jeffery S. (2000). Linkage of recessive Robinow syndrome to a 4 cM interval on chromosome 9q22. Hum. Genet. 106, 351–354 10.1007/s004390051049 10798366

[b2] Afzal A. R. Rajab A. Fenske C. D. Oldridge M. Elanko N. Ternes-Pereira E. Tüysüz B. Murday V. A. Patton M. A. Wilkie A. O. (2000b). Recessive Robinow syndrome, allelic to dominant brachydactyly type B, is caused by mutation of ROR2. Nat. Genet. 25, 419–422 10.1038/78107 10932186

[b3] Ali B. R. Jeffery S. Patel N. Tinworth L. E. Meguid N. Patton M. A. Afzal A. R. (2007). Novel Robinow syndrome causing mutations in the proximal region of the frizzled-like domain of ROR2 are retained in the endoplasmic reticulum. Hum. Genet. 122, 389–395 10.1007/s00439-007-0409-0 17665217

[b4] Bacino C. (1993). ROR2-Related Robinow Syndrome. GeneReviews Pagon R A Bird T D Dolan C R Stephens K Adam M P, ed: University of Washington,

[b61] Bakker E. R. Raghoebir L. Franken P. F. Helvensteijn W. van Gurp L. Meijlink F. van der Valk M. A. Rottier R. J. Kuipers E. J. van Veelen W. Smits R. (2012). Induced Wnt5a expression perturbs embryonic outgrowth and intestinal elongation, but is well-tolerated in adult mice. Dev. Biol. 369, 91–100 10.1016/j.ydbio.2012.06.007 22691362

[b5] Butler M. G. Wadlington W. B. (1987). Robinow syndrome: report of two patients and review of literature. Clin. Genet. 31, 77–85 10.1111/j.1399-0004.1987.tb02773.x 3549067 PMC5493386

[b6] Carreira-Barbosa F. Concha M. L. Takeuchi M. Ueno N. Wilson S. W. Tada M. (2003). Prickle 1 regulates cell movements during gastrulation and neuronal migration in zebrafish. Development 130, 4037–4046 10.1242/dev.00567 12874125

[b7] Curtin J. A. Quint E. Tsipouri V. Arkell R. M. Cattanach B. Copp A. J. Henderson D. J. Spurr N. Stanier P. Fisher E. M. (2003). Mutation of Celsr1 disrupts planar polarity of inner ear hair cells and causes severe neural tube defects in the mouse. Curr. Biol. 13, 1129–1133 10.1016/S0960-9822(03)00374-9 12842012

[b8] Deans M. R. Antic D. Suyama K. Scott M. P. Axelrod J. D. Goodrich L. V. (2007). Asymmetric distribution of prickle-like 2 reveals an early underlying polarization of vestibular sensory epithelia in the inner ear. J. Neurosci. 27, 3139–3147 10.1523/JNEUROSCI.5151-06.2007 17376975 PMC6672483

[b9] Etheridge S. L. Ray S. Li S. Hamblet N. S. Lijam N. Tsang M. Greer J. Kardos N. Wang J. Sussman D. J. (2008). Murine dishevelled 3 functions in redundant pathways with dishevelled 1 and 2 in normal cardiac outflow tract, cochlea, and neural tube development. PLoS Genet. 4, e1000259 10.1371/journal.pgen.1000259 19008950 PMC2576453

[b10] Fanto M. McNeill H. (2004). Planar polarity from flies to vertebrates. J. Cell Sci. 117, 527–533 10.1242/jcs.00973 14730010

[b11] Gao B. Song H. Bishop K. Elliot G. Garrett L. English M. A. Andre P. Robinson J. Sood R. Minami Y. (2011). Wnt signaling gradients establish planar cell polarity by inducing Vangl2 phosphorylation through Ror2. Dev. Cell 20, 163–176 10.1016/j.devcel.2011.01.001 21316585 PMC3062198

[b12] González-Sancho J. M. Brennan K. R. Castelo-Soccio L. A. Brown A. M. (2004). Wnt proteins induce dishevelled phosphorylation via an LRP5/6- independent mechanism, irrespective of their ability to stabilize beta-catenin. Mol. Cell. Biol. 24, 4757–4768 10.1128/MCB.24.11.4757-4768.2004 15143170 PMC416421

[b13] Hamblet N. S. Lijam N. Ruiz-Lozano P. Wang J. Yang Y. Luo Z. Mei L. Chien K. R. Sussman D. J. Wynshaw-Boris A. (2002). Dishevelled 2 is essential for cardiac outflow tract development, somite segmentation and neural tube closure. Development 129, 5827–5838 10.1242/dev.00164 12421720

[b14] Ho H. Y. Susman M. W. Bikoff J. B. Ryu Y. K. Jonas A. M. Hu L. Kuruvilla R. Greenberg M. E. (2012). Wnt5a-Ror-Dishevelled signaling constitutes a core developmental pathway that controls tissue morphogenesis. Proc. Natl. Acad. Sci. USA 109, 4044–4051 10.1073/pnas.1200421109 22343533 PMC3306699

[b15] Jenny A. Mlodzik M. (2006). Planar cell polarity signaling: a common mechanism for cellular polarization. Mt. Sinai J. Med. 73, 738–750. 17008934

[b16] Jenny A. Reynolds-Kenneally J. Das G. Burnett M. Mlodzik M. (2005). Diego and Prickle regulate Frizzled planar cell polarity signalling by competing for Dishevelled binding. Nat. Cell Biol. 7, 691–697 10.1038/ncb1271 15937478

[b17] Karner C. M. Chirumamilla R. Aoki S. Igarashi P. Wallingford J. B. Carroll T. J. (2009). Wnt9b signaling regulates planar cell polarity and kidney tubule morphogenesis. Nat. Genet. 41, 793–799 10.1038/ng.400 19543268 PMC2761080

[b18] Kibar Z. Vogan K. J. Groulx N. Justice M. J. Underhill D. A. Gros P. (2001). Ltap, a mammalian homolog of Drosophila Strabismus/Van Gogh, is altered in the mouse neural tube mutant Loop-tail. Nat. Genet. 28, 251–255 10.1038/90081 11431695

[b19] Kibar Z. Torban E. McDearmid J. R. Reynolds A. Berghout J. Mathieu M. Kirillova I. De Marco P. Merello E. Hayes J. M. (2007). Mutations in VANGL1 associated with neural-tube defects. N. Engl. J. Med. 356, 1432–1437 10.1056/NEJMoa060651 17409324

[b20] Liu C. Wang Y. Smallwood P. M. Nathans J. (2008). An essential role for Frizzled5 in neuronal survival in the parafascicular nucleus of the thalamus. J. Neurosci. 28, 5641–5653 10.1523/JNEUROSCI.1056-08.2008 18509025 PMC6670808

[b21] Liu C. Bakeri H. Li T. Swaroop A. (2012). Regulation of retinal progenitor expansion by Frizzled receptors: implications for microphthalmia and retinal coloboma. Hum. Mol. Genet. 21, 1848–1860 10.1093/hmg/ddr616 22228100 PMC3313798

[b22] Liu C. Lin C. Whitaker D. T. Bakeri H. Bulgakov O. V. Liu P. Lei J. Dong L. Li T. Swaroop A. (2013). Prickle1 is expressed in distinct cell populations of the central nervous system and contributes to neuronal morphogenesis. Hum. Mol. Genet. 22, 2234–2246 10.1093/hmg/ddt075 23420014 PMC3652420

[b23] May-Simera H. Kelley M. W. (2012). Examining planar cell polarity in the mammalian cochlea. Methods Mol. Biol. 839, 157–171 10.1007/978-1-61779-510-7_13 22218900

[b24] Montcouquiol M. Rachel R. A. Lanford P. J. Copeland N. G. Jenkins N. A. Kelley M. W. (2003). Identification of Vangl2 and Scrb1 as planar polarity genes in mammals. Nature 423, 173–177 10.1038/nature01618 12724779

[b25] Morgan D. Turnpenny L. Goodship J. Dai W. Majumder K. Matthews L. Gardner A. Schuster G. Vien L. Harrison W. (1998). Inversin, a novel gene in the vertebrate left-right axis pathway, is partially deleted in the inv mouse. Nat. Genet. 20, 149–156 10.1038/2450 9771707

[b26] Murdoch J. N. Doudney K. Paternotte C. Copp A. J. Stanier P. (2001). Severe neural tube defects in the loop-tail mouse result from mutation of Lpp1, a novel gene involved in floor plate specification. Hum. Mol. Genet. 10, 2593–2601 10.1093/hmg/10.22.2593 11709546

[b27] Narimatsu M. Bose R. Pye M. Zhang L. Miller B. Ching P. Sakuma R. Luga V. Roncari L. Attisano L. (2009). Regulation of planar cell polarity by Smurf ubiquitin ligases. Cell 137, 295–307 10.1016/j.cell.2009.02.025 19379695

[b28] Ovchinnikov D. (2009). Alcian blue/alizarin red staining of cartilage and bone in mouse. Cold Spring Harb. Protoc. 2009, pdb prot5170 10.1101/pdb.prot5170 20147105

[b29] Patton M. A. Afzal A. R. (2002). Robinow syndrome. J. Med. Genet. 39, 305–310 10.1136/jmg.39.5.305 12011143 PMC1735132

[b30] Peng Y. Axelrod J. D. (2012). Asymmetric protein localization in planar cell polarity: mechanisms, puzzles, and challenges. Curr. Top. Dev. Biol. 101, 33–53 10.1016/B978-0-12-394592-1.00002-8 23140624 PMC4854750

[b31] Qian D. Jones C. Rzadzinska A. Mark S. Zhang X. Steel K. P. Dai X. Chen P. (2007). Wnt5a functions in planar cell polarity regulation in mice. Dev. Biol. 306, 121–133 10.1016/j.ydbio.2007.03.011 17433286 PMC1978180

[b32] Schlessinger K. Hall A. Tolwinski N. (2009). Wnt signaling pathways meet Rho GTPases. Genes Dev. 23, 265–277 10.1101/gad.1760809 19204114

[b33] Seifert J. R. Mlodzik M. (2007). Frizzled/PCP signalling: a conserved mechanism regulating cell polarity and directed motility. Nat. Rev. Genet. 8, 126–138 10.1038/nrg2042 17230199

[b34] Simons M. Gloy J. Ganner A. Bullerkotte A. Bashkurov M. Krönig C. Schermer B. Benzing T. Cabello O. A. Jenny A. (2005). Inversin, the gene product mutated in nephronophthisis type II, functions as a molecular switch between Wnt signaling pathways. Nat. Genet. 37, 537–543 10.1038/ng1552 15852005 PMC3733333

[b35] Sinha T. Wang B. Evans S. Wynshaw-Boris A. Wang J. (2012). Disheveled mediated planar cell polarity signaling is required in the second heart field lineage for outflow tract morphogenesis. Dev. Biol. 370, 135–144 10.1016/j.ydbio.2012.07.023 22841628 PMC3432663

[b36] Takeuchi M. Nakabayashi J. Sakaguchi T. Yamamoto T. S. Takahashi H. Takeda H. Ueno N. (2003). The prickle-related gene in vertebrates is essential for gastrulation cell movements. Curr. Biol. 13, 674–679 10.1016/S0960-9822(03)00245-8 12699625

[b37] Tao H. Suzuki M. Kiyonari H. Abe T. Sasaoka T. Ueno N. (2009). Mouse prickle1, the homolog of a PCP gene, is essential for epiblast apical-basal polarity. Proc. Natl. Acad. Sci. USA 106, 14426–14431 10.1073/pnas.0901332106 19706528 PMC2732806

[b38] Tao H. Inoue K. Kiyonari H. Bassuk A. G. Axelrod J. D. Sasaki H. Aizawa S. Ueno N. (2012). Nuclear localization of Prickle2 is required to establish cell polarity during early mouse embryogenesis. Dev. Biol. 364, 138–148 10.1016/j.ydbio.2012.01.025 22333836 PMC3299875

[b39] Tissir F. Qu Y. Montcouquiol M. Zhou L. Komatsu K. Shi D. Fujimori T. Labeau J. Tyteca D. Courtoy P. (2010). Lack of cadherins Celsr2 and Celsr3 impairs ependymal ciliogenesis, leading to fatal hydrocephalus. Nat. Neurosci. 13, 700–707 10.1038/nn.2555 20473291

[b40] van Amerongen R. Fuerer C. Mizutani M. Nusse R. (2012). Wnt5a can both activate and repress Wnt/β-catenin signaling during mouse embryonic development. Dev. Biol. 369, 101–114 10.1016/j.ydbio.2012.06.020 22771246 PMC3435145

[b41] Veeman M. T. Slusarski D. C. Kaykas A. Louie S. H. Moon R. T. (2003). Zebrafish prickle, a modulator of noncanonical Wnt/Fz signaling, regulates gastrulation movements. Curr. Biol. 13, 680–685 10.1016/S0960-9822(03)00240-9 12699626

[b42] Vladar E. K. Bayly R. D. Sangoram A. M. Scott M. P. Axelrod J. D. (2012). Microtubules enable the planar cell polarity of airway cilia. Curr. Biol. 22, 2203–2212 10.1016/j.cub.2012.09.046 23122850 PMC3518597

[b43] Wallingford J. B. (2006). Planar cell polarity, ciliogenesis and neural tube defects. Hum. Mol. Genet. 15, R227–R234 10.1093/hmg/ddl216 16987888

[b44] Wallingford J. B. Harland R. M. (2002). Neural tube closure requires Dishevelled-dependent convergent extension of the midline. Development 129, 5815–5825 10.1242/dev.00123 12421719

[b45] Wallingford J. B. Fraser S. E. Harland R. M. (2002). Convergent extension: the molecular control of polarized cell movement during embryonic development. Dev. Cell 2, 695–706 10.1016/S1534-5807(02)00197-1 12062082

[b46] Wang Y. Nathans J. (2007). Tissue/planar cell polarity in vertebrates: new insights and new questions. Development 134, 647–658 10.1242/dev.02772 17259302

[b47] Wang Y. Guo N. Nathans J. (2006). The role of Frizzled3 and Frizzled6 in neural tube closure and in the planar polarity of inner-ear sensory hair cells. J. Neurosci. 26, 2147–2156 10.1523/JNEUROSCI.4698-05.2005 16495441 PMC6674805

[b48] Wang Y. Chang H. Nathans J. (2010). When whorls collide: the development of hair patterns in frizzled 6 mutant mice. Development 137, 4091–4099 10.1242/dev.057455 21062866 PMC2976288

[b49] Wang B. Sinha T. Jiao K. Serra R. Wang J. (2011). Disruption of PCP signaling causes limb morphogenesis and skeletal defects and may underlie Robinow syndrome and brachydactyly type B. *Hum.* Mol. Genet. 20, 271–285 10.1093/hmg/ddq462 PMC3031336 20962035

[b50] Watanabe D. Saijoh Y. Nonaka S. Sasaki G. Ikawa Y. Yokoyama T. Hamada H. (2003). The left-right determinant Inversin is a component of node monocilia and other 9+0 cilia. Development 130, 1725–1734 10.1242/dev.00407 12642479

[b51] Wu J. Roman A. C. Carvajal-Gonzalez J. M. Mlodzik M. (2013). Wg and Wnt4 provide long-range directional input to planar cell polarity orientation in Drosophila. Nat. Cell Biol. 15, 1045–1055 10.1038/ncb2806 23912125 PMC3762953

[b52] Yamaguchi T. P. Bradley A. McMahon A. P. Jones S. (1999). A Wnt5a pathway underlies outgrowth of multiple structures in the vertebrate embryo. Development 126, 1211–1223. 10021340 10.1242/dev.126.6.1211

[b53] Yang T. Bassuk A. G. Fritzsch B. (2013). Prickle1 stunts limb growth through alteration of cell polarity and gene expression. Dev. Dyn. 242, 1293–1306 10.1002/dvdy.24025 23913870 PMC3985166

[b54] Yates L. L. Papakrivopoulou J. Long D. A. Goggolidou P. Connolly J. O. Woolf A. S. Dean C. H. (2010). The planar cell polarity gene Vangl2 is required for mammalian kidney-branching morphogenesis and glomerular maturation. Hum. Mol. Genet. 19, 4663–4676 10.1093/hmg/ddq397 20843830 PMC2972698

[b55] Yu H. Smallwood P. M. Wang Y. Vidaltamayo R. Reed R. Nathans J. (2010). Frizzled 1 and frizzled 2 genes function in palate, ventricular septum and neural tube closure: general implications for tissue fusion processes. Development 137, 3707–3717 10.1242/dev.052001 20940229 PMC2964100

[b56] Zheng L. Zhang J. Carthew R. W. (1995). frizzled regulates mirror-symmetric pattern formation in the Drosophila eye. Development 121, 3045–3055. 7555730 10.1242/dev.121.9.3045

