# Supplementary material for: Null and hypomorph Prickle1 alleles in mice phenocopy human Robinow syndrome and disrupt signaling downstream of Wnt5a
Source: Biol Open. 2014 Sep 4;3(9):861–70. doi: 10.1242/bio.20148375 (PMC4163663; doi:10.1242/bio.20148375)
Supplement: Supplementary Material [file supp_3_9_861__index.html]

Null and hypomorph Prickle1 alleles in mice phenocopy human Robinow syndrome and disrupt signaling downstream of Wnt5a — Null and hypomorph Prickle1 alleles in mice phenocopy human Robinow syndrome and disrupt signaling downstream of Wnt5a — Supplementary Material 

# Null and hypomorph *Prickle1* alleles in mice phenocopy human Robinow syndrome and disrupt signaling downstream of Wnt5a

## bio.20148375 Supplementary Material

**Files in this Data Supplement:**

- Supplementary Material - Chunqiao Liu et al. doi: 10.1242/bio.20148375
