# Supplementary material for: Null and hypomorph Prickle1 alleles in mice phenocopy human Robinow syndrome and disrupt signaling downstream of Wnt5a
Source: Biol Open. 2014 Sep 4;3(9):861–70. doi: 10.1242/bio.20148375 (PMC4163663; doi:10.1242/bio.20148375)
Supplement: Supplementary Material [file supp_bio.20148375_bio.20148375-s1.pdf]

Supplementary Material
Chunqiao Liu et al. doi: 10.1242/bio.20148375

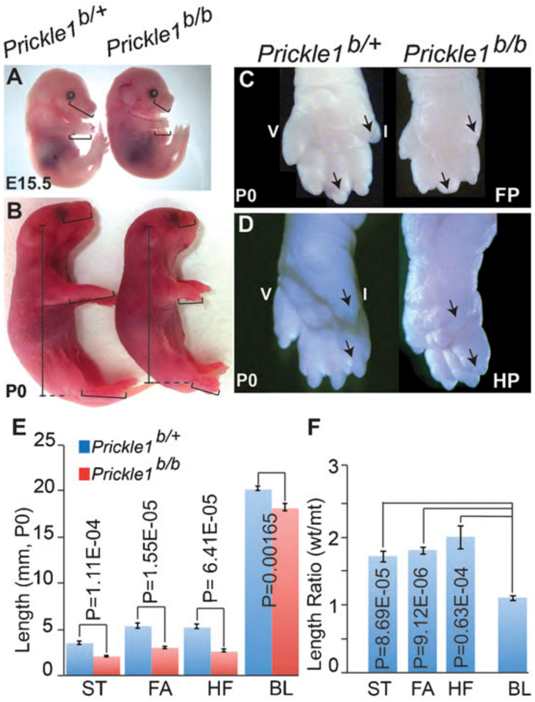

Fig. S1. Shortened limb and snout in *Pk1* mutants. (A) E15.5 embryos. Brackets indicate the length of snout and forearm. (B) P0 pups. Brackets indicate the length of snout, forearm and hind foot. Vertical line segments starting from above ear to the base of the tail indicate the body length (BL). (C,D) A close view of fore- and hind- paws showed brachydactly of mutant fingertips. FP, forepaw; HP, hindpaw. Arrows point to fingertips.
(E) Quantification of the length of snout (ST, measured from eye to the tip of the nose), forearm (FA, measured from the elbow of the forelimb), hind foot (HF) and body axis (BL). Five wild type and mutant P0 pups were measured. (F) Ratios of wild type and mutant snout, forearm and hind foot in comparison with the ratio of body length. Student t-test was used to detect P values.

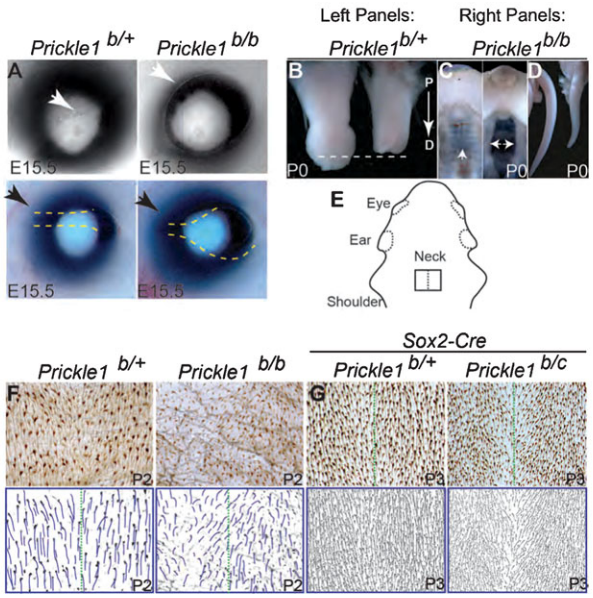

Fig. S2. Eyelid closure, tongue shape, palate fusion, and tail. Abnormalities. (A) Delayed eyelid closure *Prickle1* mutant embryos at E15.5 (Note the clearer view of mutant lens from the open eyelid). (B) Truncated and misshapen tongue of a *Prickle1* mutant pup at P0. (C) *Prickle1* mutant pup showed cleft palate defect. Arrows point to the fusion edges of the palates. (D) Shortened and curvy mutant tails. (E) Drawing of a top view of the head of a P3. Squared area is the location that hair images were taken. The hair follicles at this location (shoulder level) are relatively more developed than the posterior areas at this age. (F) Mutant hair follicles are less development and less organized at P2. Lower panels are tracings of the hair follicle orientations from the image above. Dashed lines mark the midlines. (G) $Pk1^{b/c}$ conditional mutants in Sox2-Cre transgenic background showing midline hair development and orientation defects at P3.

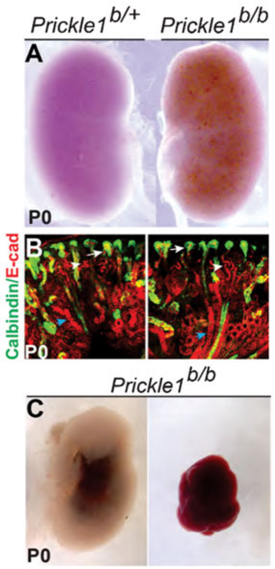

Fig. S3. Kidney appearance and tubule development. (A) Blood spots shown on the surface of the mutant kidney. (B) Cabindin-28K stained ureteric bud (green, white arrows) and E-cadherin (red) stained distal convoluted tubules (arrowheads) and ureteric collecting ducts (red, turquoise arrows). Ureteric buds and collecting ducts develop normally at gross anatomic level. (C) Cystic-like appearance of mutant kidneys in rare cases at P0 (3 out of 61).

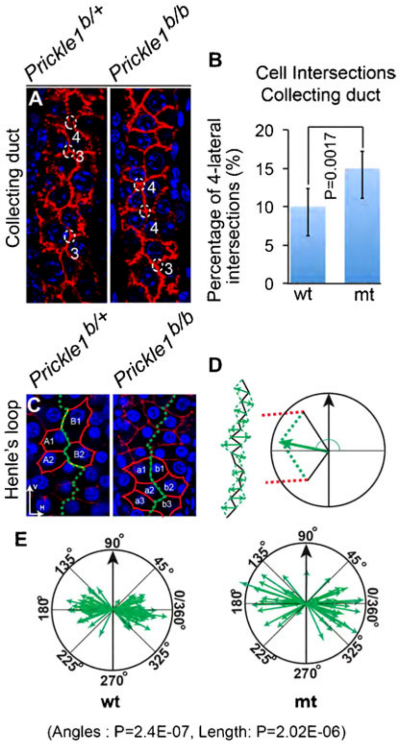

Fig. S4. Cell connection and intercalation mode is altered in the renal tubules and collecting duct. (A) Longitudinal view of collecting ducts from the renal medulla area by E-cadherin staining. (B) Quantification of cell connection mode showing increased in 4-lateral connections in the mutant epithelium. (C) Cell arrangement in the ascending Henle's loop visualized by E-cadherin staining. Dotted green lines are junctions of neighboring two columns of cells indicated respectively by 'A/B' and 'a/b' in the wild types and the mutants along vertical axis. Arrows indicate vertical and horizontal axes. (D) Schematic illustration of cell laterals vectorial addition. A shortest compound line path (in black, left) was defined between two adjacent columns of cells along the vertical direction of a tubule (also dotted green lines in C). The vectorial additions were performed for laterals from each cell vertex on the compound line. (E) Collections of acquired vectors were plotted in a polar coordinate. Paired t-test analysis was performed for relative lengths and angles measured referring to the horizontal axis using ImageJ. Angles, P=2.4E-07; Lengths: P=2.02E-06. 68 vectors acquired about equally from three animals of either wild type or the mutants were analyzed plotted.

Biology Open (2014) 000, 1–10 doi:10.1242/bio.20148375

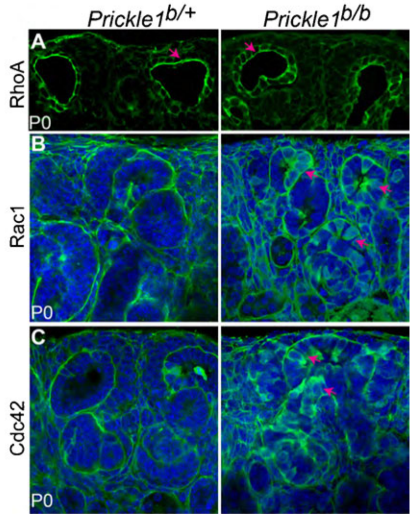

Fig. S5. Ectopic deposition of Rac1 and Cdc42 and mislocalization of actin in chondrocytes. (A) RhoA staining. Arrows indicate apical localization of RhoA. (B) Rac1 staining. (C) Cdc42 staining. Arrows indicate the ectopic staining of Rac1 and Cdc42 in the mutant tissue.

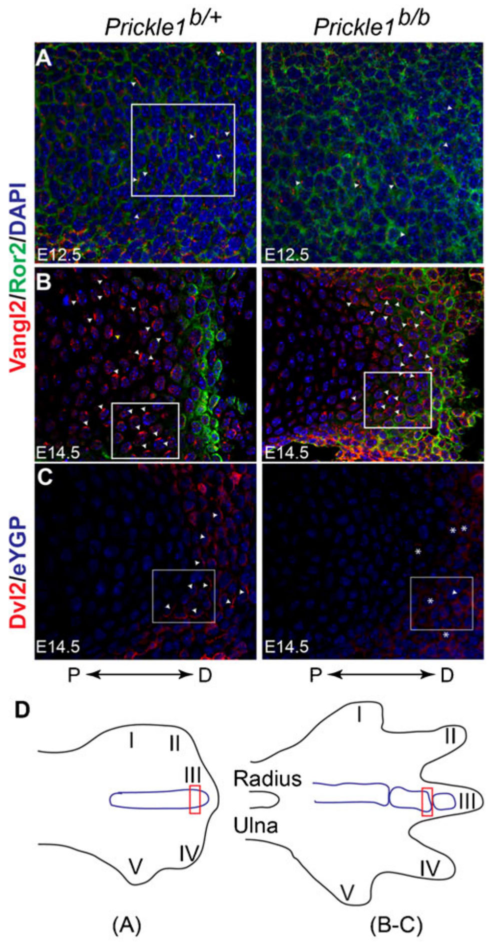

Fig. S6. Vangl2 and Dvl2 localization in the developing chondrocytes. (A) Polarized Vangl2 (Red) staining in both wild type and the mutant digit chondrocytes. Ror2 staining (green) did not show a polarized pattern. Boxed areas are zoomed in Fig. 7A. (B) Generally retained Vangl2 (red) polarization at E14.5 digit bones. Boxed areas are zoomed in Fig. 7B. Yellow arrow labeled an oppositely polarized cell near the ossification center. (C) Loss of Dvl2 polarization in the mutant chondrocytes (asterisks). Boxed areas are zoomed in Fig. 7C. (D) Schematic drawing of the locations of the images taken at E12.5 (Left panel) and E14.5 (right panel) forelimb.

Biology Open

Biology Open (2014) 000, 1–10 doi:10.1242/bio.20148375

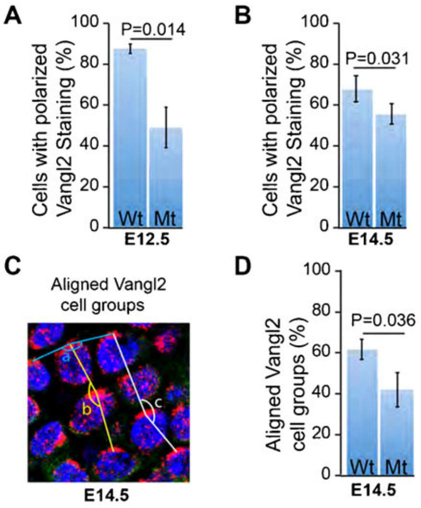

Fig. S7. Quantifications of alignment of Vangl2 staining. (A,B) Percentage of polarized Vangl2 chondrocytes is decreased in Prickle1. A: E12.5; B, E14.5. (C) Illustration of defining the aligned cell groups. Alignment is considered only if three center points of Vangl2 staining make an angle equal or large than 165 degree (arbitrarily defined), for example angles a, b and c. (D) Percentage of aligned cell groups in both wild type and mutant digit tissue. Three wild type and mutant embryos were analyzed, each of which 24 cell groups were analyzed.

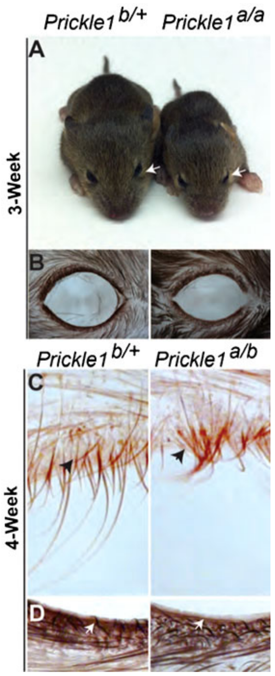

Fig. S8. Eye lashes defects in *Prickle1* a/a and *Prickle1* a/b, hypomorphic mutant mice. All mutants are shown in right panels. (A) 3-week old wild type and $Pk1^{a/a}$ mutant pair. Arrows point abnormal eyelids/ eyelashes. (B) Skin preparation for a close view of eyelids and eyelashes. (C) High magnification of upper eyelashes of 4-week old $Prickle1^{a/b}$ mice. (D) lowered lower eyelashes in the mutant (arrows).

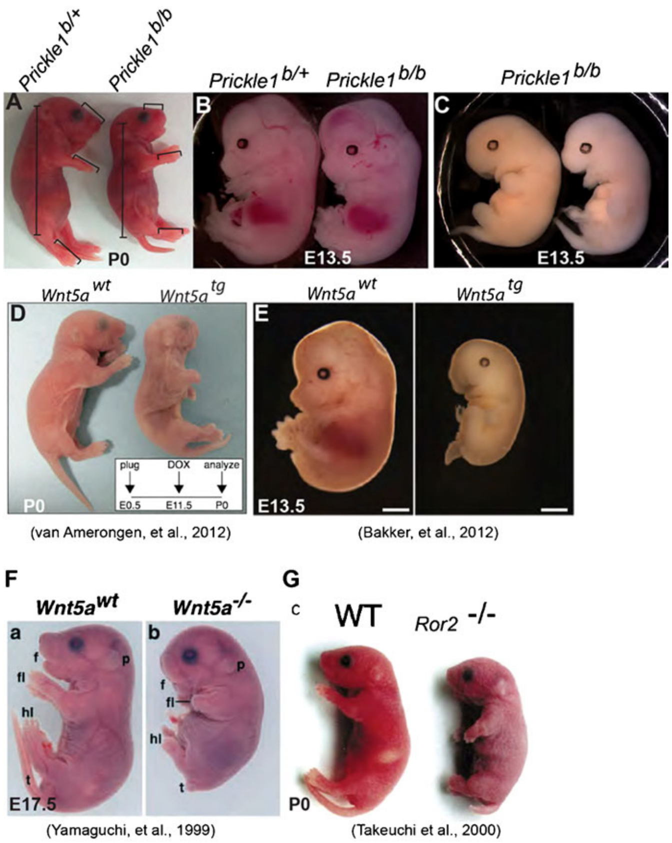

Fig. S9. A comparison of gross morphology of $Pk1^{b/b}$ mutants with that of *Wnt5a* signaling pathway mutations. (A) $Pk1^{b/b}$ mutants show tissue distal truncation defects at P0. (B) E13.5 mutant embryos showing shorted forelimb and less pointed nose. (C) In rare cases, embryos die early at E13.5 showing less developed all distal structures. (D) Inducible *Wnt5a* transgenic mice showing similar morphology to that of $Pk1^{b/b}$ mutants at P0 (A) (van Amerongen et al., 2012. (E) Severer morphological defects in Wnt5a overexpression at E13.5 (Bakker et al., 2012). (F) $Wnt5a^{-/-}$ mutant embryos at E17.5 (Yamaguchi et al., 1999). (G) $Ror2^{-/-}$ mutant embryos at P0 (Takeuchi et al., 2003).

Biology Open

Biology Open (2014) 000, 1–10 doi:10.1242/bio.20148375

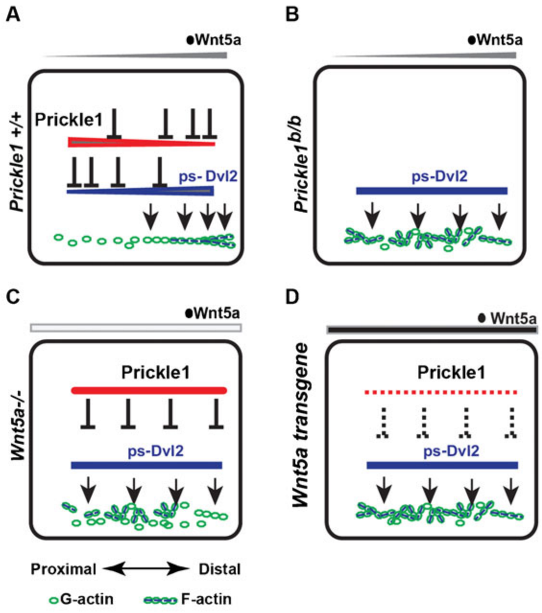

Fig. S10. A working model for Wnt5a signaling driven asymmetrical actin assembly. (A) In wild type cells, Wnt5a gradient creates a Prickle1 gradient through promotion of Prickle1 degradation along proximal-distal axis. Prickle1 gradient further facilitates the formation of a reverse gradient of phosphorylated form of Dvl2 to direct asymmetrical actin assembly. (B) Loss of Prickle1 will disrupt ps-Dvl2 gradient along distal-proximal axis leading to randomized actin assembly. (C) In *Wn5a* null mutants, loss of Wnt5a (therefore its gradient) lowers phosphorylation of Dvl2 and disrupts its gradient formation. Consequently, actin assembly is hindered. (D) In *Wn5a* transgenic mice, overexpression of Wn5a overrides its gradient formation. At the same time, the overdose of Wnt5a signaling causes Prickle1 degradation. Therefore, the effect on ps-Dvl2 gradient caused by *Wnt5a* overexpression is similar to that by *Prickle1* ablation as described in B. Arrows stand for positive regulation. Inversed ‘‘T’’s stand for inhibition. Dotted ‘‘T’’s stand for less inhibition. Shallow triangles indicate gradients.

Biology Open
